# How, when, and who should ask about pregnancy intentions in primary care? A qualitative study of primary healthcare professionals’ preferences

**DOI:** 10.3399/BJGPO.2024.0148

**Published:** 2024-11-27

**Authors:** Jennifer Hall, Helen Carr, Anne Connolly, Geraldine Barrett

**Affiliations:** 1 Sexual and Reproductive Health Research Team, Elizabeth Garrett Anderson Institute for Women’s Health, University College London, London, UK; 2 NHS Surrey Heartlands, Surrey, UK; 3 Bevan Healthcare, Bradford, UK

**Keywords:** pregnancy intention screening, preconception health and care, contraception, pregnancy, reproductive care, primary health care

## Abstract

**Background:**

Knowing people’s pregnancy intentions would help healthcare professionals (HCPs) to take a more holistic approach to reproductive health and preconception care.

**Aim:**

To assess the feasibility of implementation of questions about pregnancy preferences in a range of primary care settings in Great Britain, including digital implementation.

**Design & setting:**

Qualitative study using online semi-structured interviews with primary healthcare professionals across Great Britain between February and July 2022.

**Method:**

Twelve online interviews were conducted with GPs (*n* = 3), practice nurses (*n* = 3), sexual and reproductive health professionals (*n* = 4), and health visitors (*n* = 2). Framework analysis was conducted in NVivo, adapting a coding frame from complementary interviews with women.

**Results:**

HCPs perceived asking about pregnancy preferences as valuable in meeting patients’ reproductive health needs and most suited to women’s health consultations, medication and disease reviews, baby checks, or as an addition to lifestyle questions leading to health promotion advice. An introductory, non-discriminatory signposting sentence was well-liked, and the preferred question in a face-to-face clinical encounter was asking how the person would feel about a pregnancy in the next year, in line with women’s preference. Guidance and training would give clinicians confidence in knowing how to ask about pregnancy preferences and advise their patients accordingly.

**Conclusion:**

Asking about pregnancy intentions is acceptable to women and HCPs and feasible in primary care, but implementation needs to be adapted to the patient and context. Digital options that enable patients to self-manage can reduce the need for HCP input and avoid medicalising a normal process.

## How this fits in

Asking about pregnancy intentions is vital to identify who would benefit from preconception advice or contraception to improve reproductive health across the life course. Healthcare professionals (HCPs) can find it hard to raise this topic because of concerns over sensitivity, and a lack of time, tools, and training. Asking about pregnancy intentions across a range of settings in primary care is acceptable and feasible to women and HCPs; how it is done needs to be adapted to the patient and context, including digital options. Training that highlights the value of intervention, addresses HCPs’ concerns about patients’ reactions, and provides evidence-based ways to sensitively raise the question of pregnancy intentions would enable provision of much-needed support to patients.

## Introduction

There is consensus in the literature that primary care is the best place for pregnancy intention screening^1,2^ and that it could be initiated by HCPs.^3,4^ Indeed, 91% of primary care practitioners thought pregnancy intention screening should be routine practice.^1^ However, HCPs lack the tools to do this effectively.^2^ In order to deliver the strategy for preconception health proposed in *The Lancet* in 2019, to *‘normalise conversations about planning for pregnancy’* and *‘improve identification of people who are planning a pregnancy’*,^5^ we need a robust and acceptable way of asking people what their preferences are.^6^ The identification of people who are thinking about, or open to, pregnancy is currently a significant barrier to the ability to provide timely preconception health advice.^7,8^ The ability to do so would enable HCPs to better meet their patients’ needs by taking a holistic approach to the reproductive lifecourse,^9–11^ opening discussion about an area that has been neglected.^12–14^

Several ways of asking about pregnancy preferences exist. These include variations of a reproductive life plan as recommended by the US Centers for Disease Control and Prevention, the One Key Question (OKQ) approach,^15^ the psychometrically validated Desire to Avoid Pregnancy (DAP) scale,^16^ and screening for family planning service needs.^1,17^ Two systematic reviews have shown these approaches to be generally acceptable to women and HCPs, and feasible to introduce in some settings,^18,19^ but there was no clear preference between them.^1,20^ However, most evidence is from the US; to the best of our knowledge, no data on the preferences of HCPs in Great Britain exists.

The UK-based Pregnancy Planning, Prevention and Preparation Study (P3 Study) explored different ways of asking about pregnancy preferences, including the DAP scale^16^ and a question like the OKQ. Based on analyses of these data, a shortlist was developed using questions from the DAP (a 14-item scale, developed to measure women’s preferences and feelings about a potential pregnancy, see Supplementary Appendix S1), of which its predictive ability with regard to pregnancy was known.^21–23^ This study aimed to assess the acceptability of the shortlist to HCPs and feasibility of its implementation in a range of primary care settings in the UK, including options for digital implementation. Women’s views have been published elsewhere.^24^

## Method

The study was designed, implemented, and reported in line with the consolidated criteria for reporting qualitative research (COREQ).^25^

### Recruitment

We aimed to recruit around 15 primary care professionals working with women of reproductive age for in-depth interviews; 2–4 from each of: GPs; practice nurses (PNs); community sexual and reproductive health (CSRH) doctors or nurses; and health visitors (HVs). HCPs were recruited through professional networks, electronic mailing lists of relevant professional groups (for example, the Institute for Health Visiting and the Faculty of Sexual and Reproductive Health), and snowball sampling (PNs only). Pharmacists were not recruited as women previously reported not wanting to be asked in a pharmacy.^10,26^

### Data collection

The topic guide (see Supplementary Appendix S2) complemented the topic guide used to explore women’s preferences^24^ and covered the specific questions, how they might be used in healthcare settings or consultations, and different format options. HCPs saw the questions on screen to give them time to consider them (Table 1). A ranking exercise was incorporated to facilitate discussion around format preferences, including digital options.

**Table 1.** Wording of questions discussed in the interviews (questions are taken from the Desire to Avoid Pregnancy scale). A five-point Likert scale ranging from ‘strongly disagree’ to ‘strongly agree’ was used.

How much would you agree or disagree that:
It would be a good thing for you if you became pregnant in the next 3 months
It would be the end of the world for you to have a baby in the next year
You want to have a baby within the next year
Thinking about becoming pregnant in the next 3 months makes you feel excited
You would worry that having a baby in the next year would make it harder for you to achieve other things in your life

### Data analysis

Our methodological approach was applied clinical qualitative research aligned with qualitative description.^27^ We conducted a framework analysis,^28^ using NVivo (version 20), focusing on experiential themes relating to the participants’ feelings and preferences.^29^ JH adapted the coding frame from the complementary interviews exploring women’s preferences,^24^ adding two new themes (HCPs’ experiences of asking about pregnancy preferences and challenges to implementation) and two sub-themes. The revised coding framework was tested on two transcripts from different professions, discussed with GB (who had access to the original interview transcripts and provided an independent, second perspective), and applied to all interviews in the indexing stage by JH. During charting, findings across the themes were explored by profession. Findings were regularly discussed, including with the patient and public involvement (PPI) group.

### Patient and public involvement

The P3 Study PPI group, comprising 20 women aged 18–44 years from across the UK, discussed the shortlisted questions, reviewed the topic guide, and gave feedback on findings during the analysis.

### Research team and reflexivity

JH, a female public health researcher with a medical background and training and experience in qualitative interviewing, conducted and recorded the interviews online over Zoom, transcribed them verbatim, and anonymised them. She is a married mother of three children with a long-standing research interest in helping people achieve their reproductive goals. Field notes were used; participants were asked if they wished to see their transcript, but none returned any comments. JH knew none of the participants before interview; participants were fully aware of the purpose of the research before providing informed consent and no one else was present during the interview.

## Results

### Sample

Twenty-two HCPs responded and 21 were eligible for interview (one was not working in the UK). We purposively selected HCPs to ensure a range of professions and geography to ensure a maximum-variation sample and increase the information power of each interview.^30^ Twelve online interviews were conducted between February and July 2022; four HCPs were invited to interview but did not book (two CSRH nurses and two HVs), and four CSRH doctors and one PN were not invited because there were already sufficient numbers in those groups. Details of HCPs, who had up to 25 years’ experience, are summarised in Table 2. Interviews lasted 50 minutes on average. Two PNs knew each other, but worked in different practices.

**Table 2.** Details of participants’ profession, location, and sex

Identifier	Occupation	Location	Sex
CSRH1	CSRH doctor (including abortion care)	North East	Female
CSRH2	CSRH doctor (including trans health)	London	Male
CSRH3	CSRH nurse (including abortion care)	North East	Female
CSRH4	CSRH doctor (including abortion care)	Wales	Female
GP1	GP	London	Male
GP2	GP	Scotland	Female
GP3	GP	Midlands	Female
HV1	Health visitor	North East	Female
HV2	Health visitor	Midlands	Female
PN1	Practice nurse	South West	Female
PN2	Practice nurse	South West	Female
PN3	Practice nurse	South East	Female

CSRH = community sexual and reproductive health.

### Findings

**Figure 1.**
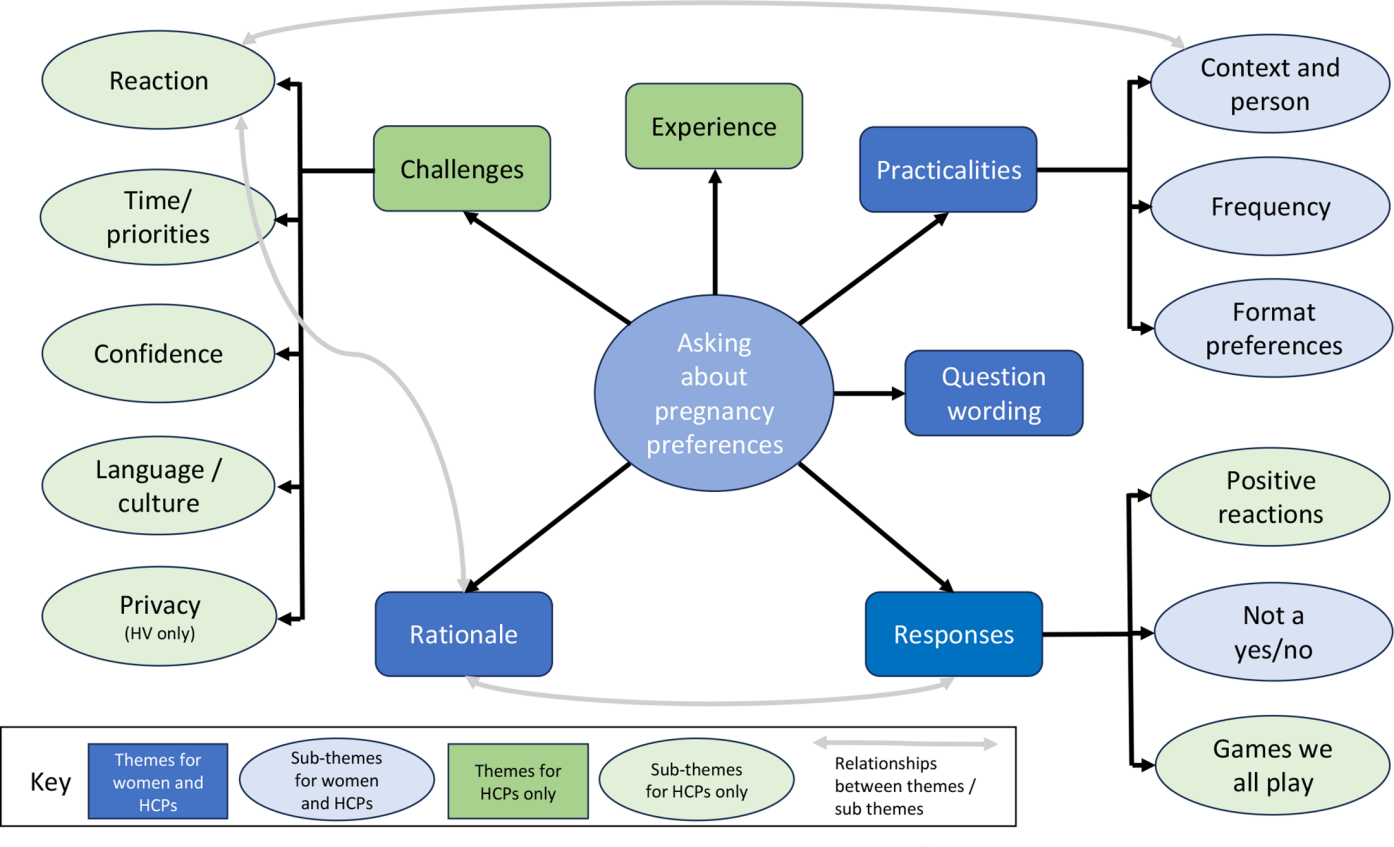
Themes and sub-themes relating to HCPs’ experience and opinions of asking patients about pregnancy preferences. HCP = healthcare professional. HV = health visitor.

The six themes, further sub-themes, and the overlap with the themes from women’s preferences,^24^ are shown in Figure 1.

#### Experience of asking about pregnancy preferences

The extent of HCPs’ experience ranged from HVs who did it frequently to PNs who did it rarely. GPs and CSRH were mixed, but one felt *‘it’s not that different to what I do already’* (CSRH4). Profession, experience, patient group, and the types of consultations they have (for example, whether or not they provide contraception) influenced HCPs’ thoughts regarding asking about pregnancy preferences and the challenges they faced.

#### Rationale for asking

The overriding reason for asking patients about their pregnancy preferences was to *‘get a feel for what are they thinking about it* [pregnancy] *… and do we as healthcare professionals need to put anything in place for them, or … signpost them somewhere’* (PN1) to ensure that they were *‘offering the right care’* (CSRH1). This was both for whether they needed preconception or contraception advice and, if the latter, to determine the best method of contraception:

*‘I think your choices* [of contraception] *are governed by what their sort of five-year plan is, so they understand that and they're very open to talk about it.’* (GP3)

#### Responses

Patients’ responses to being asked about pregnancy intentions fell into three sub-themes. First, no HCP had received a negative reaction. Indeed, GP3 described a very positive response from one person, who was glad that a rubella screen had been included with her blood tests. GP3 went on to say that people are *‘very open … as long as they understand why you’re asking, people are only too happy to tell you, no one ever says “that’s not your business’’, they’re very happy’,* demonstrating the links between rationale, response, and reaction.

Second, women’s responses were not yes/no answers; they are *‘usually a description’* (CSRH4) of their reasons, which then *‘opens up the conversation’* (HV1).

Finally, there was an understanding of *‘the games, we all play’* (GP3) and *‘the things that aren’t said in the GUM* [genitourinary medicine] *clinic’* (CSRH2). This meant that, in the HCPs’ opinion, people were not always forthcoming or completely honest in disclosing pregnancy intentions:

*‘People do, I think, say, “Oh we’re kind of trying”, or like they stopped not trying, you know by like dropping off the contraception, but maybe they’re not going to focus intensely on the, following the cycle and stuff, but maybe kind of subconsciously in a way, hoping to get pregnant.’* (CSRH2)

*‘... people sometimes like to pretend that* [they] *haven’t started trying when they have in case that they don’t get pregnant.’* (GP3)

#### Practicalities

##### Context

HCPs felt questions about pregnancy intention fitted best within ‘women’s health’ consultations and therefore within every CSRH and HV consultation, but only within selected primary care consultations. While PN3 expressed initial hesitancy around contraception consultations, she later said *‘I guess we should probably say, “Are you happy on this?” And, you know, talk about return to fertility, which would bring it all up*.*’* The PNs, who all performed smear tests, felt that this was *‘a natural area for it to go’* (PN3), and fitted well with existing questions about hormonal contraception.

Outside a ‘women’s health’ consultation, opinions were more divided. GP1 and PN1 thought the 6-week baby check and baby immunisations could be a good option, but PN3 disagreed. PN1 and PN3 thought their asthma and/or diabetes clinics could be a good opportunity, because pregnancy could affect their condition and vice versa, but noted the time constraints. GP2 thought that reviews of medications *‘that might be detrimental* [to pregnancy]*’* could be suitable, but *‘if they’ve come in for a sore throat, I’m not going to start asking them about their attitudes to pregnancy’*.

GPs felt that it could *‘definitely’* (GP2) be incorporated into routine lifestyle checks: *‘if it was kind of a sort of do ya smoke? Do ya drink? Planning a baby? I think that would be a really good thing. I don’t know why it hasn’t been done, really’* (GP3). Asking it this way could help with normalisation, as GP2 said:

*‘I don’t think it’s an embarrassing thing to ask at all … if it’s part of a routine list of questions I don’t think patients get offended by that.’* (GP2)

However, GP1 noted that they did not always have time to run through these questions.

##### Person (who should ask?)

*‘There seems to be a vacuum’*, so said HV2 of the professionals dealing with the preconception period. This was reflected by others, such as CSRH4, who noted that they would deal with those who want to avoid pregnancy but:

*‘... the people who perhaps want to get pregnant or want to get pregnant in the near future, those are the people that aren’t getting the advice … because … midwives don’t get involved until they’re 12 weeks pregnant, and then GPs are very, very busy, so who sees those people?’* (CSRH4)

CSRH1, in suggesting that people would see their GP about this, realised that this might work for people with chronic conditions:

‘... *whereas I guess if you’re a healthy person you’re probably not going to go to your GP and actually you’re more likely to get that information from someone giving you contraception.’* (CSRH1)

So, while the initial reactions of some CSRHs were that they were not the right person as *‘if someone wants to get pregnant that, that’s almost, not the end of our job, but you know ok that’s all the contraception thing we don’t have to do, let’s go on to the STIs now*’ (CSRH2), they later said, in line with CSRH1, *‘Actually we're a good place to do it.’* CSRH3 described being a *‘bridge’* because:

*‘I’m the person stopping the contraception, they’ve told me that they wish to get pregnant, so I’m telling them that they should start the folic acid, vitamin D, stop smoking, stop drinking, if they're overweight consider weight loss, things like that all the relevant things that are supporting a healthy pregnancy and hopefully fulfilling their wishes.’* (CSRH3)

CSRH2 pondered how their department would respond *‘if someone came in saying “I’m here for advice on how to optimise my fertility or advice on preparing for pregnancy”’* but CSRH3 thought *‘it is quite an old-fashioned view that if someone’s wanting to get pregnant, they shouldn’t be accessing our service*.’ PN1 summarised it as:

*‘... the reality is that I guess we’re more reactive only and “oh you’re pregnant” and we rely on somebody else, some other unknown persons, to advise that woman on pre-pregnancy care.’* (PN1)

GPs generally suggested that PNs should ask in smear, contraception, or annual disease review consultations, but seemed less keen to ask themselves, except as part of a series of lifestyle questions. All HCPs agreed that either a doctor or nurse could ask these questions. There was disagreement about the role of healthcare assistants (HCAs), with GP3 suggesting that HCAs could chat with patients while taking blood as patients do not have an agenda, but PN3 felt that HCAs would not be able to give the patients the information they need and so should not ask.

### Challenges to implementation

Four challenges were noted by all specialities and one by HVs only.

#### Confidence

Two female CSRH doctors, a female CSRH nurse, a female HV, and the two female GPs felt equally confident in advising about either contraception or preconception health because they had professional and/or personal experience to draw on. Of the other six: five, including the two male HCPs and all the PNs, felt more confident in contraception (for example, *‘I wouldn’t know where to begin with preconception health’* [PN1]); and one PN did not feel particularly confident in either. Most felt their colleagues would be more comfortable in giving advice around contraception.

Therefore, a potential barrier was not wanting to raise a topic if feeling unable to advise:

*‘*... *if you don't know enough about preconception counselling or about contraception you’re kind of setting yourself up for an awkward conversation, because you go “Oh, would you like contraception?” They go, “yes, can we talk about it?” And you go “oh actually no, I don't know.”*’ (GP1)

Most noted that they had not had specific training in preconception care and felt a list or template of *‘this is preconception counselling’* (CSRH1) would help.

#### Time and competing priorities

Many commented on time pressures in their role (although CSRHs less so), either in terms of the amount to cover in a consultation or contact, or the volume of contacts. Particularly for HVs, this meant they would focus on the most pressing issue, for example, the mother’s mental health. However, HVs noted they already have some relevant skills and are well-placed to expand on pregnancy preferences. Several participants commented that once it is incorporated into your routine, time is not really an issue (CSRH2 and PN1), especially in CSRH where it is *‘fundamentally important ... it should be part of the consultation … it’s part of your care that you need to offer’* (CSRH1).

#### Concern about reactions

While none of the participants had experienced any negative reactions when asking about pregnancy intentions, there were several reasons why HCPs were wary of this. These included *‘stigma about asking people about pregnancy’* (CSRH3), and that it can be triggering for the person if they might never want children, cannot have children, have had a recent miscarriage, or for trans people. However, without wishing to deliberately upset people, it was recognised that:

*‘... if they tell you that, actually, they’ve had loads* [of] *miscarriages or they’re struggling to get pregnant, that could be the start of a conversation about that too. So … I don’t think that’s the reason not to ask the question … because it could be upsetting.’* (GP1)

#### Language and cultural issues

Language and cultural issues were raised as a barrier. This included women with English as a second language due to potential difficulties in understanding questions (especially the ‘end of the world’ question [see Table 1]). Potential issues around the cultural appropriateness of questions and the potential for differing expectations of motherhood were also raised. The use of interpreters was a further potential barrier, particularly if the interpreter was male, but also because consultations take longer and these questions may not be deprioritised.

#### Privacy (HVs only)

As HVs see women in their own homes there are often other people present. They must be skilled at getting women alone in order to ask certain things (for example, abuse) and felt that questions about future pregnancy may need to be asked in private for the woman to answer honestly.

### Question wording

HCPs’ thoughts about the specific questions are shown in Table 3.

**Table 3.** Summary of HCPs’ thoughts about each proposed question from the Desire to Avoid Pregnancy scale and their own suggestions

Question	Summary of HCPs’ thoughts
I ask all my patients of reproductive age about pregnancy, in case I can offer advice about contraception or preparation for pregnancy [is that ok?]	The suggested introduction was generally well received because it includes both pregnancy and preconception and does not single people out, therefore it is not assuming anything or discriminating. Signposting like this was seen as a recognised technique, giving permission in the moment and a chance for patients to say if they do not want to discuss it.
How much would you agree or disagree that it would be a good thing for you if you became pregnant in the next 3 months?	Best liked overall as opener (PN2) and similar to things they might already say (HV1). CSRH2 was initially unsure and thought it might be too emotive, but while talking came around to this one: ‘*Because, maybe it does kind of capture that, like it’s not my overarching purpose or ... it’s not like the one thing I’m focused on but actually I’m kind of secretly hoping it happens, and if it captures that a bit better*… [links to responses/things unsaid].’ There were some concerns about the unintended interpretation of a suggestion that the HCP thought it would be a good thing for them (GP2 and PN3), or that pregnancy is generally a good thing (CSRH1).
How much would you agree or disagree that it would be the end of the world for you to have a baby in the next year?	Generally felt to be negative, emotive, and dramatic, and some HCPs’ reaction was that they would never use it, particularly the PNs. However, many noted that patients might use this phrase (CSRH2, GP2, HV1, and CSRH3) and several HCPs had used it, for example: *‘... if you have a young fertile woman telling me she’s using condoms I would then say, “would it be the end of the world if you were pregnant?” Because if the answer is yes that’s not good enough. So yes, I do frequently in that scenario definitely ask someone about that.’* (GP3) It was recognised that it gives you an idea of how strongly someone feels about pregnancy and, like GP3, CSRH1, CSRH4, and PN1, all described using or could imagine using it in a contraceptive scenario.
How much would you agree or disagree that you want to have a baby within the next year?	Not a strong reaction to this, seen as a bit more neutral, relaxed possibly, more familiar/comfortable, but CSRH3 did not like this, did not like use of ‘want’, which felt expectational. This was the only one GP2 might contemplate asking, but even then only to an older woman or one she knew was trying to conceive, but would leave out the timeframe.
How much would you agree or disagree that thinking about becoming pregnant in the next 3 months makes you feel excited?	Generally not liked. Feels more like a *‘product user survey’* (CSRH2) not so much a question for a clinical consultation. CSRH4 thought this carried an expectation that you should feel excited about being pregnant. For GP3 it was too vague, flippant, focused on short-term excitement not long-term reality, and could be patronising to some (older) women.
How much would you agree or disagree that you would worry that having a baby in the next year would make it harder for you to achieve other things in your life?	Also generally not liked, although some reported circumstances where it could be useful. Generally did not seem like the sort of thing they would ask for a variety of reasons but some scenarios where it might be used were for: women who have had, or are at risk of having, more than one child removed from their care; teenage parents; or other patients where *‘you know from knowing them’* (GP1) that pregnancy might be challenging for them, as a way of encouraging them to think about the impacts or reality of having a(nother) baby.
How would you feel about a pregnancy in the next year?	This question was suggested independently by several women and health professionals (such as HV1). It was put to eight out of the 12 HCPs (those whose interviews happened after it had been proposed by several women) and was widely liked as more open (CSRH4 and HV2). They felt they were more likely to use this as it sounded more like a question that they would use (GP2, GP3, PN1, PN2, and PN3).
Other suggestions: *‘Is your family complete?’* was suggested by GPs and HVs when seeing a woman who already had children. Questions relating to service needs were also suggested.	

CSRH = community sexual and reproductive health. HCP = healthcare professional. HV = health visitor. PN = practice nurse.

When considering how many questions to ask in a face-to-face setting, flexibility and adaptation to context and client were key. As emphasised by several HCPs:

*‘... in terms of … the best outcomes for the client and understanding their needs, it’s better to ask as many questions and … have as thorough a conversation as you can.’* (HV1)

*‘... when it comes to a patient in front of you it actually does matter quite a lot to them whether they get pregnant or not.’* (CSRH1)

While the DAP questions can give the HCP a probability of pregnancy in the next year, HCPs were unlikely to use this during opportunistic screening. The DAP questions ask about a 3-month or a 1-year time frame. PN1 and CSRH2 liked 3 months as *‘it gives you the idea that someone’s got an immediate desire, next 3 months, basically means, in my mind, that’s “I want to get pregnant now”’* (CSRH2), but for some 3 months felt quite *‘pressurised’* (PN2). For HVs, the preferred time frame depended on how postnatal their client was. CSRH4’s usual time frame to ask was a year, mainly because of return of fertility after contraceptive injections. However, some did not like or use a timeframe (GP2, GP3, and PN3).

### Frequency and target population

Asking females annually from 16 or 18 years of age was considered reasonable, if not necessarily feasible either as a result of capacity or lack of contact. There were concerns about groups for whom these questions could be triggering. This included trans people, although CSRH2, who works in a gender clinic, felt that it was still important to ask, probably at the first meeting:

*‘I don’t think you should be scared to ask, initially, because of course trans people are thinking about this as well.’* (CSRH2)

Using the introduction to signpost what is coming, allowing the patient to close the conversation, and asking in a sensitive way were key to addressing this.

### Format preferences

When considering whether these questions should be asked in person or digitally (by text message or on a website/app) there was a definite sense that it was *‘not a case of one size fits all’* (PN3) and that there should be flexibility to use a different format, approach, or question(s) in different scenarios:

*‘.*.. *because some people, I think, prefer to read things and then ask questions. Whereas with other people … they might not have even thought about half of it if you don't bring it up as a conversation, so I think a combination and it depends on the person.’* (HV1)

Digital options would usually be self-complete, making using several DAP questions and calculating the probability of pregnancy more feasible.

Overall, there was a preference for asking digitally first for several reasons: reluctance to medicalise this life stage; the fact this applies to a generally healthy population that is not frequently seen in person; the changing context of primary care, with much more digital interaction and increasing e-health literacy among patients; that patients would prefer it; plus time pressures and other priorities. However, as a traditionally signposting service, and perhaps because HVs are unlikely to be in triggering situations, HV1 was more comfortable with asking the questions and directing people to online or other advice.

## Discussion

### Summary

Across a range of primary care professionals, asking about pregnancy intentions, while not routine practice, was seen as relevant and feasible. Opinions on the wording of specific questions varied according to the types of consultations and patient population. While whether pregnancy would be a ‘good thing’ was most liked from the DAP questions, in a face-to-face clinical setting there was an overall preference for the question suggested and preferred by women^24^ of ‘How would you feel about a pregnancy in the next year?’ as a more open question that allowed patients to elaborate. Narrow questions, such as ‘Are you currently trying for a baby?’, do not take account of the fact that people’s thoughts and plans may be fluid or vague, or they may not feel comfortable expressing such certainty. This contributes to the perception that answers to questions about pregnancy are not always completely truthful, and to the preference of HCPs and women^24^ for a more open question.

The options of asking in person, digital tools for self-management, and digital approaches from the health service were all considered viable and could be adopted and adapted to different settings. Many primary care consultations are now digital first, which facilitates asking about pregnancy intentions and sending the patient links to more detailed information or advice. While an in-person opportunistic screening may raise the topic with an open question, digital or self-complete options might contain the DAP questions to identify more accurately who is likely to become pregnant. This could be particularly relevant in the management of pre-existing conditions.

### Strengths and limitations

A particular strength of this study is the diverse range of HCPs included, most of whom did not have a particular interest in the area of preconception health. However, a limitation is that only two were male, in part a reflection of the workforce. Since the two male practitioners reported feeling less confident in preconception advice, further work with this group might be warranted. While diverse in clinical practice and locations, all HCPs were native English speakers. Language and cultural issues may affect these conversations, or attitudes to having them, which would be worth further exploration. The complementary nature of the study on women’s preferences is novel in that we have explored preferences from both sides of the patient–professional relationship. It is important that pregnancy intention screening is inclusive, as neither sex nor gender determine someone’s childbearing intentions, and anyone who could contribute to the conception of a child should be encouraged to consider contraception or preconception health.

### Comparison with existing literature

There was uncertainty as to who should be responsible for asking about pregnancy preferences in primary care, in keeping with other literature.^7,12,14,31^ CSRH HCPs often came to the realisation during the interview that they were ideally placed, and women preferred CSRH professionals to be asking them.^24^ Asking about pregnancy intentions in CSRH is in line with the Hatfield Vision^32^ of discussing preconception health in every contraception consultation. However, CSRH services are sparse and most contraception is prescribed in general practice,^33^ making it essential that GPs and PNs are engaged; women were also comfortable speaking to these HCPs.^10,24^

There are strong precedents in primary care for ‘Making Every Contact Count’ (MECC)^34^ and the value of *‘very brief advice’* for smoking cessation,^35^ both of which are highly relevant to screening for pregnancy intentions. Consultations that are for women’s health conditions, disease management or medication review, or that are for a practical procedure like a smear can be used, with the option to signpost to digital support or printouts as the patient prefers. This is important as there would not be time for thorough discussion given that this is a conversation tacked on to another agenda. This MECC approach is acceptable to women on the condition that they understand the rationale for asking.^24^ The target population for these conversations are generally healthy and have little routine contact with health services, which can be a further barrier.^12^ Digital methods, such as targeted text messages, plus social media campaigns and inclusion in relationships and sex education in secondary school, as suggested by younger women,^24^ would enhance reach. However, in recommending digital approaches it is important to consider the digital divide and to ensure that other formats remain available to prevent increases in health inequalities.

HCPs raised potential barriers including time pressures, concerns over reactions, and confidence in giving advice, particularly on preconception health, as other studies have found.^12,31^ With regards to time, it was noted that it simply needed to be incorporated into a routine; the fact that it could be implemented digitally also addresses this concern. As previously noted,^24^ further work exploring the preferences of underserved groups, including trans and non-binary people and migrants, and co-producing approaches and materials that are culturally sensitive are important next steps. A good example of where this has already occurred is the ‘Starting Well’ project in west Yorkshire, which delivers preconception care tailored for the most underserved groups in society, including refugees, sex workers, and people experiencing homelessness.^36^

### Implications for practice

Many HCPs had received little training in preconception health, and our 2020 assessment of the preconception content of various undergraduate and postgraduate medical curricula showed that it was lacking (Hanson *et al*, unpublished data, 2022). Regardless, most were aware of important topics to discuss, suggesting that the issue is not solely a lack of knowledge but a lack of familiarity with applying this health promotion information to the population of reproductive age. Where pregnancy intention screening has been trialled, and combined with HCP training, there is some evidence of increases in knowledge and changes in practice, but less of changes in behaviours or outcomes.^37,38^ To overcome this, training that highlights the value of preconception intervention, in addition to the content, would help HCPs understand why it is important and the impact that their brief intervention can have. Training can also address HCPs’ concerns about patients’ reactions by reassuring them of the evidence behind the advised approach, including its acceptability, and that while it might be upsetting for some patients this is not a reason not to ask, provided it is done sensitively, as this could enable provision of much-needed support to the patient. Suggestions for how to raise the topic in different settings are shown in Table 4.

**Table 4.** Suggested wording and settings for pregnancy intention screening, based on findings from the interviews in the present study, and suggestions made by women and healthcare professionals^24^

In clinical settings: GPs, practice nurses, and community sexual and reproductive health doctors and nurses
**Opportunistic screening in clinical settings**
Any women’s health related consultation, for example, smear, contraception, menstrual issues, and/or sexual health
At a review for an existing condition, for example, asthma, diabetes, epilepsy, and/or thyroid disease
When prescribing or reviewing medication
As part of lifestyle checks when asking about smoking or alcohol consumption, for example
At postnatal, 6-week baby check or baby immunisation appointments, or any health visitor contact, for those who already have a child
**Suggested wordings**
Introductory (signposting) sentence that may be sufficient in some cases: I ask all my patients of reproductive age about pregnancy, in case I can offer advice about contraception or preparation for pregnancy. Is that something you would be interested in?
Would a pregnancy in the next year be a good thing for you? Which could be followed up, if needed with: Would it be the end of the world?
How would you feel about a pregnancy in the next year?
If you are thinking of trying for a baby or need contraception, feel free to mention it and we can have a chat
Would you like to discuss pregnancy or contraception? If you're thinking of pregnancy in the next few years, it would be really good to discuss this
Health visitors, or where the healthcare professional knows the patient already has at least one child: Is your family complete?
**In education settings**
In secondary schools and further education settings: incorporation of fertility awareness, reproductive life planning, contraception, and messages around the importance of preparing for pregnancy, if that is desired in the future, into relationships, sex, and health education. Opening questions could include: Do you want children in your life? How do you plan to prevent becoming pregnant until you are ready? Would you like to learn about ways to prepare for a healthy pregnancy in the future?
In higher education: information during fresher’s fairs and at university health services, linked with information on consent and sexually transmitted infections
**Digital**
Direct approaches from the health service, for example, text messages to selected sub-groups of the registered population at a practice; better availability of online resources, such as websites or apps, which could contain the Desire to Avoid Pregnancy questions

Asking about pregnancy intentions across a range of settings in primary care is acceptable and feasible, but how it is done needs to be adapted to the patient and context. Despite recognition among HCPs of the significance and value of these discussions, many are unsure how to sensitively broach the topic, untrained in the advice to give, and unable to see how to incorporate it into already overwhelmed systems, demonstrating the need for guidance and training. While there are ways to incorporate pregnancy intention screening into consultations this is not always necessary and may serve to unnecessarily medicalise a normal part of the reproductive life course. Digital formats need to be promoted for those who are not in contact with HCPs so as to avoid increasing inequalities. Greater awareness among HCPs, educational settings, and wider society will help to normalise these discussions and facilitate a more holistic approach to reproductive health across the life course.

## Data

The data underlying this article cannot be shared publicly as we do not have consent to place transcripts in a public repository. The anonymised transcripts will be shared on reasonable request to the corresponding author.
